# Exploring the size of reference population for expected accuracy of genomic prediction using simulated and real data in Japanese Black cattle

**DOI:** 10.1186/s12864-021-08121-z

**Published:** 2021-11-06

**Authors:** Masayuki Takeda, Keiichi Inoue, Hidemi Oyama, Katsuo Uchiyama, Kanako Yoshinari, Nanae Sasago, Takatoshi Kojima, Masashi Kashima, Hiromi Suzuki, Takehiro Kamata, Masahiro Kumagai, Wataru Takasugi, Tatsuya Aonuma, Yuusuke Soma, Sachi Konno, Takaaki Saito, Mana Ishida, Eiji Muraki, Yoshinobu Inoue, Megumi Takayama, Shota Nariai, Ryoya Hideshima, Ryoichi Nakamura, Sayuri Nishikawa, Hiroshi Kobayashi, Eri Shibata, Koji Yamamoto, Kenichi Yoshimura, Hironori Matsuda, Tetsuro Inoue, Atsumi Fujita, Shohei Terayama, Kazuya Inoue, Sayuri Morita, Ryotaro Nakashima, Ryohei Suezawa, Takeshi Hanamure, Atsushi Zoda, Yoshinobu Uemoto

**Affiliations:** 1 National Livestock Breeding Center, Nishigo, Fukushima 961-8511 Japan; 2 Livestock Research Institute, Animal Research Center, Hokkaido Research Organization, Shintoku, Hokkaido 081-0038 Japan; 3 Aomori Prefectural Industrial Technology Research Center, Tsugaru, Aomori, 038-2816 Japan; 4 Iwate Agicultural Research Center Animal Industry Research Institute, Takizawa, Iwate 020-0605 Japan; 5 Miyagi Prefecture Animal Industry Experiment Station, Osaki, Miyagi 989-6445 Japan; 6 Akita Prefectural Livestock Experiment Station, Daisen, Akita 019-1701 Japan; 7 Livestock Research Centre, Fukushima Agricultural Technology Centre, Fukushima, 960-2156 Japan; 8 Hida Beef Cattle Research Department, Gifu Prefectural Livestock Research Institute, Takayama, Gifu 506-0101 Japan; 9 Tottori Prefectural Livestock Research Center, Kotoura, Tottori, 689-2503 Japan; 10 Shimane Prefecture Livestock Technology Center, Izumo, Shimane 693-0031 Japan; 11 Institute of Animal Production Okayama Prefectural Technology Center for Agriculture, Forestry and Fisheries, Misaki, Okayama, 709-3401 Japan; 12 Hiroshima Prefectural Technology Research Institute, Livestock Technology Research Center, Shobara, Hiroshima, 727-0023 Japan; 13 Yamaguchi Prefectural Agriculture and Forestry General Technology Center, Mine, Yamaguchi 759-2221 Japan; 14 Saga Prefectural Livestock Experiment Station, Takeo, Saga 849-2305 Japan; 15 Nagasaki Prefectural Beef Cattle Improvement Center, Hirado, Nagasaki, 859-4824 Japan; 16 Oita Prefectural Agriculture, Forestry, and Fisheries Research Center, Takeda, Oita, 878-0201 Japan; 17 Miyazaki Livestock Research Institute, Takaharu, Miyazaki 889-4411 Japan; 18 Cattle Breeding Development Institute of Kagoshima Prefecture, Soo, Kagoshima, 899-8212 Japan; 19 Okinawa Prefectural Livestock and Grassland Research Center, Nakijin, Okinawa 905-0426 Japan; 20 Genetics Hokkaido Association, Sapporo, Hokkaido 060-0004 Japan; 21 Research and Development Group, Zen-noh Embryo Transfer Center, Kamishihoro, Hokkaido 080-1407 Japan; 22 Graduate School of Agricultural Science, Tohoku University, Sendai, Miyagi 980-8572 Japan

**Keywords:** Accuracy of genomic prediction, Size of reference population, Independent chromosome segments

## Abstract

**Background:**

Size of reference population is a crucial factor affecting the accuracy of prediction of the genomic estimated breeding value (GEBV). There are few studies in beef cattle that have compared accuracies achieved using real data to that achieved with simulated data and deterministic predictions. Thus, extent to which traits of interest affect accuracy of genomic prediction in Japanese Black cattle remains obscure. This study aimed to explore the size of reference population for expected accuracy of genomic prediction for simulated and carcass traits in Japanese Black cattle using a large amount of samples.

**Results:**

A simulation analysis showed that heritability and size of reference population substantially impacted the accuracy of GEBV, whereas the number of quantitative trait loci did not. The estimated numbers of independent chromosome segments (*M*_*e*_) and the related weighting factor (*w*) derived from simulation results and a maximum likelihood (ML) approach were 1900–3900 and 1, respectively. The expected accuracy for trait with heritability of 0.1–0.5 fitted well with empirical values when the reference population comprised > 5000 animals. The heritability for carcass traits was estimated to be 0.29–0.41 and the accuracy of GEBVs was relatively consistent with simulation results. When the reference population comprised 7000–11,000 animals, the accuracy of GEBV for carcass traits can range 0.73–0.79, which is comparable to estimated breeding value obtained in the progeny test.

**Conclusion:**

Our simulation analysis demonstrated that the expected accuracy of GEBV for a polygenic trait with low-to-moderate heritability could be practical in Japanese Black cattle population. For carcass traits, a total of 7000–11,000 animals can be a sufficient size of reference population for genomic prediction.

**Supplementary Information:**

The online version contains supplementary material available at 10.1186/s12864-021-08121-z.

## Background

Genomic evaluation in beef cattle breeds have been implemented worldwide using high-density single nucleotide polymorphism (SNP) arrays [1–4], and more accurate prediction of genomic estimated breeding values (GEBVs) can promote genetic improvement in these populations. In general, the accuracy of genomic prediction of GEBVs depends on the extent of linkage disequilibrium (LD) between quantitative trait loci (QTLs) and SNPs on high-density SNP arrays in each breed [5], because the SNP arrays are designed to function for several breeds [6–10]. Thus, accuracy of genomic prediction is important to evaluate in target breed populations.

Japanese Black cattle comprise the major source of beef in Japan, and they have traditionally been bred with a focus on carcass traits, such as fat marbling. The intensive use of a few elite bulls over the years has led to a reduction in genetic diversity within the breed, and Nomura et al. [11] estimated an effective population size (*N*_*e*_) of 17.2 during 1997 using the pedigree information. In contrast, the *N*_*e*_ was much larger in other breeds. For example, one study estimated *N*_*e*_ of Angus and Hereford as being 207 and 185, respectively [12], and another estimated those of Angus and Charolais as being 207 and 285, respectively [13]. From the perspective of *N*_*e*_, the genetic structure of Japanese Black cattle is quite different from that of other beef cattle breeds; thus, the extent of the LD between QTLs and SNPs in Japanese Black cattle might differ from that of other cattle breeds.

The effectiveness of genomic evaluation for carcass traits [14, 15], the fatty acid composition of meat [16], and feed efficiency traits [17] has been assessed in Japanese Black cattle. For example, Takeda et al. [17] conducted a genomic evaluation using the genotypes of 300 bulls and the phenotypes of their progenies as a reference population and found moderate prediction reliability for feed efficiency traits. Onogi et al. [15] used various sizes and compositions for the reference population and concluded that the accuracy of genomic prediction of carcass traits could be improved by expanding the genotyped population. However, the number of animals with genotypes and trait variation used in these studies is limited. Uemoto et al. [18] conducted a genomic evaluation using simulated data accounting for the extent of LD between QTL and SNPs in Japanese Black cattle and found that size of reference population was the most important factor affecting accuracy of genomic prediction. A simulation study conducted by Takeda et al. [17] included reference populations of different sizes with a genetic structure mimicking the *N*_*e*_ of Japanese Black cattle. They also found that the size of the reference population noticeably influenced accuracy of genomic prediction. However, verification using real data has not been performed.

A study of genomic evaluation on a larger scale than previous related studies may lead to better understanding on the impact of the size of reference population on accuracy of GEBV for not only carcass traits that have been emphasized up to the present but also simulated traits. The finding might offer an insight into making decisions regarding the size of reference population in other numerically small breeds. In the current study, more than 14,000 samples from various regions in Japan were analyzed. We aimed to explore the size of the reference population for expected accuracy of GEBVs for simulated and real data in Japanese Black cattle. Firstly, we conducted a simulation analysis based on a cross-validation design using real genotypes to account for the extent of LD in Japanese Black cattle. In second, we empirically determined the expected accuracy of the GEBV using a maximum likelihood (ML) approach based on the simulation results. In third, we then investigated differences of accuracy between the expected and actual carcass traits in the same population.

## Methods

### Animals and carcass traits

Approval from the Animal Care and Use Committee was not obtained for this study, because all tissue samples for DNA extraction and carcass data were collected from cattle that had been shipped to slaughterhouses in Japan where were cared for and slaughtered according to Japanese animal welfare regulations.

We obtained data from 14,821 cattle that had been fattened in the Japanese prefectures of Hokkaido, Aomori, Iwate, Miyagi, Akita, Fukushima, Gifu, Tottori, Shimane, Okayama, Hiroshima, Yamaguchi, Saga, Nagasaki, Oita, Miyazaki, Kagoshima and Okinawa between 2007 and 2020. The mean age (± standard deviation [SD]) at the time of slaughter was 28.9 ± 1.8 months. Carcass weight (CW, kg) was defined as the sum of the left and right sides of chilled carcasses. The rib-eye area (REA, cm^2^) and subcutaneous fat thickness (SFT, cm) were measured at the sixth and seventh rib sections. The rib thickness (RT, cm) was measured at the midpoint of the seventh rib section. The beef marbling score, which was ranked from 1 (poor) to 12 (abundant), was measured at the surface of the longissimus thoracis muscle between the sixth and seventh ribs according to the Japan Meat Grading Association [19]. We transformed beef marbling scores (BMS) from 1 to 12, to 0–5 using the conversion criteria described by Oyama [20] to ensure normal distribution.

### Genotypic data, data editing, and extent of LD

Genomic DNA samples were extracted from perirenal adipose tissue using the automated nucleic acid isolation systems NA-3000 and GENE PREP STAR PI-480 (Kurabo, Osaka, Japan). The DNA of all samples genotyped using the GeneSeek Genomic Profiler: GGP BovineLD v4.0, which contained 30,105 SNPs (Illumina, San Diego, CA, USA) is described herein as SNP_LD_. We clustered SNPs using the standard cluster file distributed by Illumina Inc. and called genotypes using GenomeStudio version 2.0.5 (Illumina, San Diego, CA, USA). We excluded animals with call rate of individual < 0.95, which left 14,783 animals. The SNP positions in the array were updated to the ARS-UCD 1.2 assembly using the UCSC Genome Browser tool (http://hgdownload.soe.ucsc.edu/goldenPath/bosTau9/liftOver/), and the missing genotype of SNP_LD_ was then imputed using Beagle 5.1 software [21]. The SNP_LD_ were imputed into BovineHD BeadsChip (Illumina) using Beagle 5.1 software [20] based on the ARS-UCD 1.2 assembly. The reference population for imputation comprised the BovineHD genotypes of 1368 Japanese Black cattle [22]. These imputed SNPs are referred to herein as SNP_HD_ and were included in the simulation analysis.

We cross-validated simulated and actual carcass traits on the same level as the size of reference population by firstly editing the structure of animals and the genotypic data based on genetic relatedness and carcass records. We assessed the quality control of SNP_LD_ and SNP_HD_ using PLINK software [23], then excluded SNPs with sex chromosomes, a minor allele frequency (MAF) < 0.01, call rate of SNP < 0.95, and Hardy-Weinberg equilibrium *p* < 0.001. To avoid having close relatives and to reduce genetic bias within the population, animals with large off-diagonal elements in the genomic relationship matrix (GRM) using SNP_LD_ were removed using GCTA software [24]. The cut-off value for off-diagonal elements was set at 0.4, and 12,619 animals remained. Among carcass traits, animals with at least one trait with a value that was mean ± 3 SDs were removed. Thereafter, 12,328 animals with 18,903 SNPs on SNP_LD_ and 387,653 SNPs on SNP_HD_ remained, and Table 1 shows the distribution of these samples in feedlots by prefecture.

**Table 1:** Distribution of samples by prefecture for feedlot

Prefecture	Animals (n)	Ratio (%)
Gifu	2358	19.1%
Hiroshima	1404	11.4%
Kagoshima	1017	8.2%
Miyagi	881	7.1%
Tottori	871	7.1%
Iwate	836	6.8%
Aomori	734	6.0%
Akita	610	4.9%
Okayama	567	4.6%
Hokkaido	514	4.2%
Miyazaki	445	3.6%
Shimane	436	3.5%
Fukushima	372	3.0%
Okinawa	357	2.9%
Yamaguchi	332	2.7%
Nagasaki	293	2.4%
Oita	256	2.1%
Saga	45	0.4%
Total	12,328	100%

We estimated the LD value (*r*^*2*^), which is a measure of LD, using the SNP_HD_ of the 12,328 animals, for all pairs of SNPs < 1 Mb apart using PLINK software [23]. Average *r*^*2*^ values for a given intermarker distance, with marker distances grouped in 50 kbp bins, were estimated for each autosome. The mean *r*^*2*^ values among chromosomes were then calculated.

### GBLUP evaluation

We predicted GEBVs by the genomic best linear unbiased (GBLUP) method using the following model:

1 $$ \mathbf{y}={\mathbf{1}}_{\mathbf{n}}\upmu +\mathbf{Xg}+\mathbf{e}, $$

where **y** is a vector of phenotypic values, **1**_**n**_ is a vector of *n*, which is the number of animals, *μ* is the mean, **g** is the genomic breeding value with $\mathbf{g}\sim N\left(0,\mathbf{G}{\upsigma}_{\mathrm{g}}^2\right)$, **X** is the design matrix for **g**, **e** is the residual effect with $\mathbf{e}\sim N\left(0,\mathbf{I}{\upsigma}_{\mathrm{e}}^2\right)$; ${\upsigma}_{\mathrm{g}}^2$ and ${\upsigma}_{\mathrm{e}}^2$ are the additive genetic and residual variances, respectively, **I** is an identity matrix, and **G** is the GRM always based on the SNP_LD_ generated by the following formula [25]:

$$ \mathbf{G}=\frac{\mathbf{ZZ}^{\prime }}{\sum_{j=1}^m2{p}_j\left(1-{p}_j\right)}, $$

where *p*_*j*_ is the frequency of the second allele (A2) of the *j-th* SNP and *m* is the number of SNP_LD_ (namely 18,903). The elements of **Z** were obtained as follows:

$$ {z}_{ij}={w}_{ij}-2{p}_j, $$

where *w*_*ij*_ is the number of the second allele of animal *i* at the *j-*th SNP, which is coded as 0, 1, or 2 for the homozygote (A1A1), heterozygote (A1A2), or other homozygote (A2A2), respectively. When calculating the GRM, we added 0.00001 to the diagonal elements of each one to avoid near singularity problems. We predicted the GEBVs by incorporating the calculated GRM with SNP_LD_ using ASReml 4.1 software [26].

### Simulation analysis

We simulated the true breeding value (TBV) and phenotypes under different scenarios by varying QTL heritability and the number of QTLs. To account for the extent of the LD between QTL and SNPs in Japanese Black cattle, SNPs with MAF > 0.05 in the SNP_HD_ but not in the SNP_LD_, were randomly selected from all autosomal chromosomes and were considered as candidate QTLs. Almost all complex traits in cattle are generally assumed to have polygenic effects, and we set QTLs of 100, 500, or 2000 and three QTL heritabilities of 0.1, 0.3, or 0.5. The QTL effects were generated from a gamma distribution with shape and scale parameters of 0.4 and 1.66 [27], respectively, and signs of QTL effects were randomly selected. The phenotypic value represented the sum of the total QTL effects and the residual effect as follows:

$$ {y}_i={\sum}_{j=1}^{nQTL}{w}_{ij}{\beta}_j+{\varepsilon}_i, $$

where *nQTL* is the number of QTLs, *w*_*ij*_ is the SNP genotype for the *j*-th QTL of animal *i*, which is coded as 0, 1, or 2 for homozygote, heterozygote, or other homozygote, respectively, *β*_*j*_ is the allele substitution effect of the *j*-th QTL, *ε*_*i*_ is the residual effect generated from $N\left(0,{\upsigma}_{\mathrm{g}}^2\left(1/{h}^2-1\right)\right)$ of animal *i,* ${\sum}_{j=1}^{nQTL}{w}_{ij}{\beta}_j$ is the TBV, ${\upsigma}_{\mathrm{g}}^2$ is the total genetic variance of TBV, and *h*^2^ is the QTL heritability. Phenotypic variance was set to 100, and the total QTL variance was adjusted to 100 × *h*^2^ in all scenarios.

A reference test validation study was replicated 20 times under each scenario. We divided 12,328 animals into reference and test populations as follows. We randomly selected 1000 animals as the test population from these 12,328 animals, then 1000, 2000, 3000, 5000, 7000, 9000, and 11,000 animals were randomly selected as a reference population. Animals in a smaller reference population are always included in a larger population. The phenotypes of the animals in the test population were masked in each replicate, and the GEBV of the test population was predicted using model (1). The genetic and residual variances were fixed to predict the GEBV in each replicate, and the setting variances in each simulation scenario were used. After predicting the GEBV, the accuracy of GEBV for simulated traits was determined using Pearson’s correlation coefficients between TBVs and GEBVs. The mean ± SD of 20 replicates was obtained for each scenario and population size.

### Expected accuracy of GEBV from simulated data

A limitation of the present study is that GEBV could be predicted using a reference population of up to 11,000 animals. To estimate the accuracy of GEBVs for the simulated traits using a larger reference population, we utilized the formula suggested by Erbe et al. [28] and modified from Daetwyler et al. [28] as follows:

2 $$ r=w\bullet \sqrt{\frac{N{h}^2}{N{h}^2+{M}_e}}, $$

where *r* is the correlation coefficient between TBV and GEBV (accuracy of GEBV), *w* is the maximum accuracy of GEBV when the size of reference population is infinite at 0 ≤ *w* ≤ 1, *N* is the number of animals in the reference population, and *h*^*2*^ is the heritability of the trait, and *M*_*e*_ is the number of independently segregating chromosome segments that depends on the effective population size of the target population [29]. This model provided a perfect fit for the realized accuracy of genomic prediction in a dairy cattle population [28].

The accuracy of GEBV (*r*) in the *i*-th size of reference population in the *j*-th replicate in the simulation study was defined as *r*_*ij*_, and *r*_*ij*_ was assumed to be in normal distribution as follows:

$$ {r}_{ij}\sim N\left(E\left({r}_i\right),{\sigma}_i^2\right), $$

where *E*(r_i_) and ${\sigma}_i^2$ are respectively, the predicted value and variance of *r*_*ij*_ in the *i*-th size of reference population. We calculated the most appropriate estimates of *w* and *M*_*e*_ using the log-likelihood function as follows:

$$ \mathrm{L}\left(w,{M}_e\right)\propto -{\sum}_{i=1}^{n_{pop}}{\sum}_{j=1}^{n_{rep}}\frac{{\left\{{r}_{ij}-E\left({\mathrm{r}}_{\mathrm{i}}\right)\right\}}^2}{2{\sigma}_i^2}, $$

where *n*_*pop*_ is 7, which is the number of different size of reference population, *n*_*rep*_ is the number of replicates, namely 20, *r*_*ij*_ is the calculated accuracy of GEBVs obtained in the *i*-th size of reference population in the *j*-th replicate in each simulation scenario, and *E*(r_i_) is the predicted accuracy of GEBV determined by using model (2) and the empirical data (the setting values of *N* and *h*^*2*^ in each scenario). We assumed that ${\sigma}_i^2$ was the empirical variance in 20 replicated values within the *i-th* size of reference population in each scenario. The two parameters (*w* and *M*_*e*_) used in *E*(r_i_) were empirically determined in each scenario using the ML approach under the restriction of *w* (0 ≤ *w* ≤ 1) using the *optim* function in R software (http://www.r-project.org) for a two-dimensional search.

### Real data analysis

The variance components of carcass traits were estimated by ASReml 4.1 software [26] using the following single-trait animal model:

3 $$ \mathbf{y}={\mathbf{X}}_{\mathbf{1}}\mathbf{b}+{\mathbf{X}}_{\mathbf{2}}\mathbf{g}+\mathbf{e}, $$

where **y** is a vector of the observations; **b** is a vector of fixed effects due to prefecture for feedlot (18 classes), sex (2 classes), year of slaughter (13 classes), and covariates for age at the time of slaughter (linear and quadratic), **g** is a vector of genomic breeding values with $\mathbf{g}\sim N\left(\mathbf{0},\mathbf{G}{\upsigma}_{\mathrm{gc}}^2\right)$, where **G** and ${\upsigma}_{\mathrm{gc}}^2$ are the GRM generated with the SNP_LD_, as in model (1) and the additive genetic variance, respectively; **X**_**1**_ and **X**_**2**_ are the design matrices relating observations to fixed and random effects, respectively; **e** is a vector of residual effects with $\mathbf{e}\sim N\left(\mathbf{0},\mathbf{I}{\upsigma}_{\mathrm{e}}^2\right)$, where ${\upsigma}_{\mathrm{e}}^2$ is the residual variance.

The adjusted phenotypes (**y**_adj_) were derived by:

$$ {\mathbf{y}}_{\mathrm{adj}}=\hat{\mathbf{g}}+\hat{\mathbf{e}}, $$

where $\hat{\mathbf{g}}$ and $\hat{\mathbf{e}}$ are the predicted values of the genomic breeding value and residual effect obtained in model (3), respectively. The design of the reference-test validation study was the same as that of the simulation analysis, and model (1) was used to predict GEBV using the adjusted phenotype. The genetic and residual variances were fixed to predict the GEBV in each replicate, and we used the variance components estimated by model (3). After predicting GEBVs, their accuracy was determined using as Pearson’s correlation coefficient between the adjusted phenotypes and the GEBVs divided by the square root of the genomic heritability estimated by model (3), as described by Hayes et al. [30]. We replicated the reference-test population design 20 times for each population size, and the mean ± SD of 20 replicates was obtained.

## Results

### Linkage disequilibrium (r^2^)

Figure S1 shows the mean *r*^*2*^ for the SNP_HD_ values among chromosomes of the 12,328 animals used for analysis. Moderate linkage disequilibrium (*r*^*2*^ value = 0.2) extended to approximately 0.15 Mb.

### The accuracy of GEBV for simulated traits

**Fig. 1:**
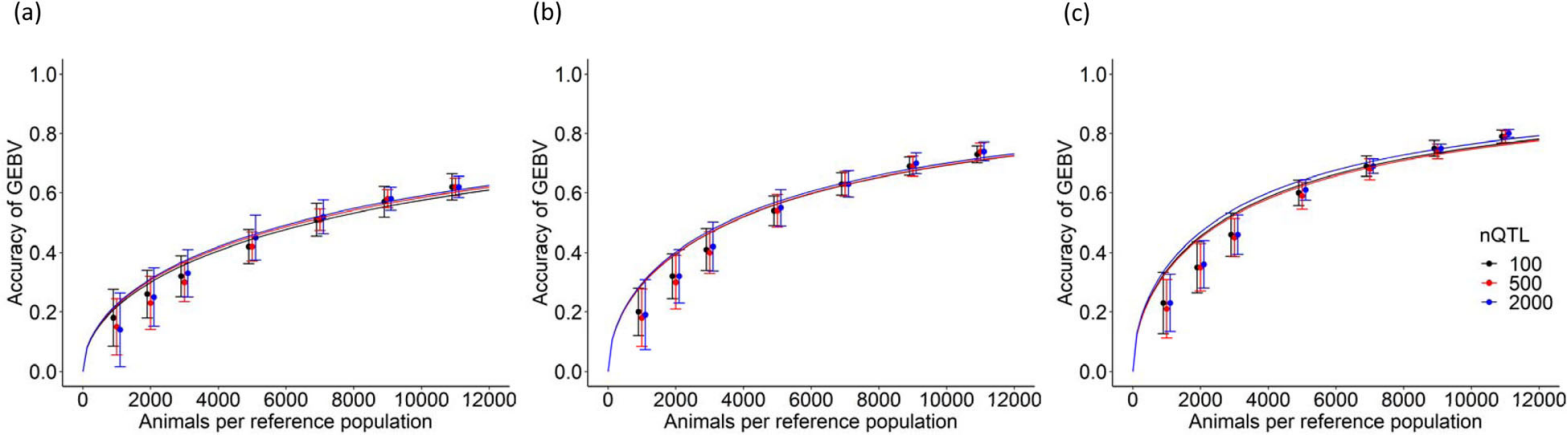
Observed and expected accuracy of genomic estimated breeding values (GEBVs) for simulated traits. Dots and curves, means of observed and predicted accuracy for simulated traits, respectively. X axis, number of animals per reference population. Y axis, observed and expected accuracy of GEBVs for simulated traits in number of QTLs (nQTL) obtained from equation developed herein. Heritability: (a), 0.1; (b), 0.3; (c), 0.5. Whiskers, standard deviations of 20 replicates for observed accuracy. The dots and error bars are intentionally staggered for clarity

Figure 1 shows the accuracy of GEBVs for predicting the simulated traits for each heritability category. Accuracy did not substantially differ according to the number of QTLs. In contrast, heritability and the size of reference population had a major impact on the accuracy. A higher value for heritability or a larger size of reference population increased the prediction accuracy of the GEBV. For example, when the QTL number was 100 and the size of reference population was 1000, the accuracy of GEBVs for heritability values of 0.1, 0.3, and 0.5 was respectively, 0.18, 0.20, and 0.23. When the reference population included 11,000 animals, the accuracy respectively improved to 0.62, 0.73, and 0.79. The SDs of GEBV accuracies decreased from ~ 0.10–0.03 as the size of reference population increased from 1000 to 11,000.

### Expected accuracy for simulated traits

Table 2 shows the estimated *M*_*e*_ values determined using the ML approach. The estimated value of *w* was 1 for all scenarios. The estimated values of *M*_*e*_ were dependent on heritability but were independent of the number of QTLs. When heritability was 0.1, 0.3, and 0.5, the estimated *M*_*e*_ values were 1900, 3200, and 3800, respectively. Figure 1 also shows the prediction accuracy of GEBVs for simulated traits (curves) in the reference population with up to 11,000 animals. Regardless of heritability, the predicted accuracy was higher than the observed accuracy for a reference population of up to 5000 animals, but came close to the observed accuracy when the reference population comprised > 7000 animals.

**Table 2:** Number of independent chromosome segments (*M*_*e*_) obtained by likelihood approach depending on condition of simulated traits

Heritability	QTL (n)	*M*_*e*_
0.1	100	2026.3
	500	1929.4
	2000	1869.2
0.3	100	3245.9
	500	3225.6
	2000	3112.4
0.5	100	3826.5
	500	3945.1
	2000	3546.2

**Fig. 2:**
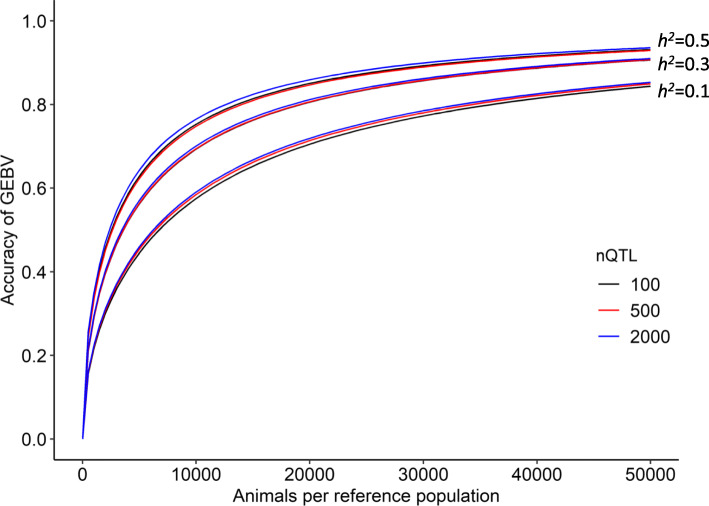
Expected accuracy of genomic estimated breeding values (GEBVs) for simulated traits. X axis, number of animals per reference population. Y axis, expected accuracy of GEBVs for simulated traits with heritability 0.1, 0.3, or 0.5 in number of QTLs (nQTL) obtained from equation developed herein

Figure 2 shows the expected accuracy of GEBVs for simulated traits due to heritability in the reference population of ≤ 50,000 animals. Values for accuracy approached 1 and approached a plateau as the reference size increased, regardless of heritability and number of QTLs. Higher heritability increased accuracy. For example, in a reference population of 20,000 animals, the estimated accuracy for the simulated traits with heritability of 0.1, 0.3, and 0.5 was respectively, 0.71, 0.81, and 0.85.

### Comparison of expected accuracy for simulated traits with accuracy for carcass traits

**Fig. 3:**
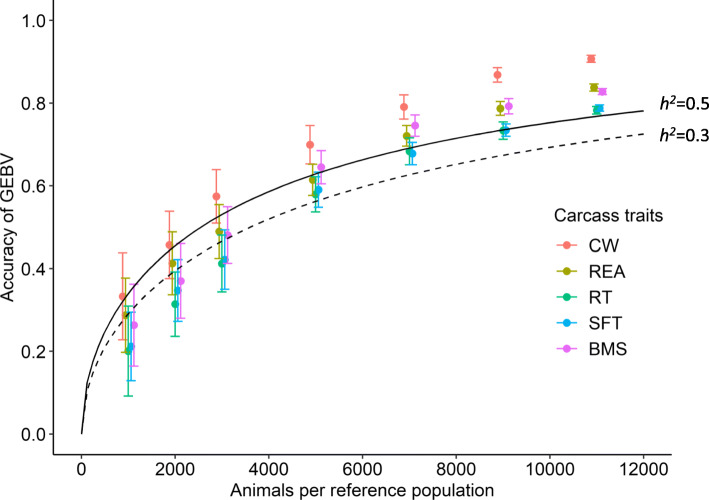
Expected accuracy of genomic estimated breeding values (GEBVs) for simulated and carcass traits. Dashed and solid curves indicate expected accuracies of GEBVs for simulated traits with 100 QTLs and heritability 0.3 and 0.5, respectively. Colored dots, means of GEBV accuracy for carcass traits. CW, carcass weight; REA, rib-eye area; RT, rib thickness; SFT, subcutaneous fat thickness; BMS, beef marbling score. Whiskers, standard deviation of 20 replicates of accuracy of GEBVs for carcass traits. X axis, number of animals per reference population. Y axis, GEBV accuracy for simulated and carcass traits. The dots and error bars are intentionally staggered for clarity

Table 3 shows descriptive statistics of carcass traits. The estimated genomic heritability of these traits was 0.29–0.41, and the estimated standard error (SE) was 0.01 for any trait. Figure 3 shows that the accuracy of GEBVs for carcass traits was 0.20–0.33 and the SD was ~ 0.1 for all traits when the reference population comprised 1000 animals. However, the accuracy range was 0.78–0.91, and the SD was < 0.01, when the reference population included 11,000 animals.

**Table 3:** Descriptive statistics of carcass traits

Trait	Mean	SD	Min	Max	Vg^b^	Ve^c^	*h*^*2* d^
Carcass weight (kg)	481.2	57.9	305.0	662.0	885.8	1278.5	0.41 (0.01)
Rib eye area (cm^2^)	62.2	10.6	32.0	97.0	37.8	58.1	0.39 (0.01)
Rib thickness (cm^2^)	8.06	0.93	5.30	10.90	0.21	0.51	0.29 (0.01)
Subcutaneous fat thickness (cm)	2.68	0.78	0.60	5.10	0.23	0.35	0.40 (0.01)
BMS^a^	2.22	0.98	0.33	5.00	0.29	0.53	0.35 (0.01)

^a^Beef marbling score

^b^Additive genetic variance accounted for by markers

^c^Residual variance

^d^Genomic heritability (strandard error)

Figure 3 compares the accuracy of genomic prediction of the simulated traits with accuracy for the carcass traits. Because the accuracy for simulated traits was not affected by the number of QTLs and the genomic heritability for carcass traits was 0.29–0.41, the accuracy in this figure is shown with 100 QTLs and heritability of 0.3 and 0.5. When the reference population comprised 11,000 animals, the expected accuracy for heritability of 0.3 and 0.5 was lower than the accuracy for all carcass traits. The accuracy for CW was much higher than the expected accuracy with a heritability of 0.5 in a reference population of > 5000 animals, considering that the estimated heritability for CW was 0.41.

## Discussion

### Importance of size of reference population to accuracy of genomic prediction

Because the LD pattern is different for each cattle population [5], it is necessary to investigate the relationship between accuracy of genomic prediction and size of reference population in a target population. We found that the LD pattern of the population used in this study differed from other beef cattle breeds [5]. Although accuracy of genomic prediction has been investigated in Japanese Black cattle [14, 15], the numbers of animals comprising the reference populations in these studies ranged from several hundred to several thousand, and the target traits were limited to carcass traits that have been emphasized in the past. In addition, the optimal number of animals in the reference population needed to further improve accuracy of genomic prediction has remained unknown. Therefore, we investigated the impact of the size of the reference population on the accuracy of genomic prediction for carcass traits using much more samples than previous studies. The SD of accuracy was < 0.01, at the maximum size of the reference population, and thus the results probably had high versatility. Onogi et al. [16] estimated heritability and the accuracy of phenotype prediction for carcass traits using the single-step GBLUP method with various sets of reference populations with up to ~ 2000 animals. Using these results, GEBV accuracy can be calculated by dividing the accuracy of phenotype prediction by the square root of the heritability estimate; for example, of 0.35–0.59 for CW and of 0.36–0.48 for BMS. Our values were consistent with these.

The degree of increase in accuracy was gentle and reached a plateau as the size of reference population increased. This agrees with previous studies of simulated [31, 32] and wheat [33] data. A critical concern is how many animals should be included in the reference population to obtain a desirable degree of accuracy of genomic prediction for carcass traits. We discuss this based on the accuracy of the conventional estimated breeding value (EBV) of a selection candidate bull progeny. Given the trait heritability (*h*^2^) and the number of progenies per candidate (*n*, half-sib), the accuracy of the EBV $\left({r}_{g,\hat{g}}\right)$ for the candidate is obtained using the general formula, ${r}_{g,\hat{g}}=\sqrt{n{h}^2/\left(4+\left(n-1\right){h}^2\right)}$ [34]. Fig. S2 shows the relationship between EBV accuracy and the number of progenies. At progeny test of candidate bulls for Japanese Black cattle, a bull is required to have a minimum of 15 progenies to obtain an EBV. Assuming 15 progenies, the accuracy of the EBVs for carcass traits ranged from 0.73 to 0.79 (Fig. S2). In addition, 7000–11,000 animals are needed, depending on the traits, in the reference population to predict GEBVs with the same accuracy as EBVs. Accordingly, when these conditions are met, the accuracy of the GEBV for carcass traits should be comparable to the EBV in the progeny test. Even slightly reduced accuracy of GEBV may be available to young candidate because long generation interval should be saved and high selection pressure can be applied. A total of 7000–11,000 animals could be a sufficient size of reference population to genetically improve carcass traits.

Japanese Black bulls have traditionally been bred on a prefectural basis for growth and meat quality and the semen of excellent bulls can be distributed in the prefecture where the bulls are produced. For example, the population in Hyogo prefecture, which is famous for Kobe beef production, has been closely bred [35]. The genetic relationship of an individual with another in the same prefecture tends to be closer than that with an individual in the other prefecture. Accordingly, when a reference and a test population are composed only of a prefecture, the accuracy of GEBV will be higher than the result of this study. This is because the accuracy of the GEBV is affected by the genetic relationship between the reference and test populations [36, 37]. Hence, the accuracy of the GEBV for an individual obtained using a country-based reference population could be lower than that of a prefecture-based reference population for specific prefectures. Further investigation is needed to address this notion, because we did not assess genetic relationships among the samples in detail.

### Simulated and expected accuracy

While our results indicated that higher heritability led to increased accuracy of genomic prediction, the number of QTLs did not. These results agree with those of a previous simulation studies [9, 18]. A larger reference population also increased accuracy of genomic prediction, which is consistent with previous studies of Japanese Black cattle [17, 18]. Although, Uemoto et al. [18] cross-validated genomic evaluation using simulated phenotypes from 1200 animals and found that accuracy of genomic prediction did not reach a plateau, the present study using the 10-fold more animals showed that accuracy of genomic prediction gradually approached a plateau.

We estimated the value of *M*_*e*_ from the accuracies empirically estimated. *M*_*e*_ is a measure of the effective number of independent segments across the genome and has been defined by various authors as a function of the historical effective population size, *N*_*e*_ (see the study of Goddard [38] for detail). The estimated *M*_*e*_ range was 1900–3900. The expected accuracy of GEBVs based on the *M*_*e*_ values were close to that obtained when the reference population contained > 5000 animals. The accuracy of GEBVs was overestimated when the reference population contained < 5000, possibly because of large deviations in observed accuracy. The *M*_*e*_ estimates obtained by empirical accuracies vary from studies and can be summarized as shown Table S1. Erbe et al. [28] estimated *M*_*e*_ of 900–2800 depending on the trait and formula in Holstein Friesian cattle and of 150–420 depending on the trait and SNP density in Brown Swiss cattle, based on cross-validation accuracies. Van den Berg et al. [39] also performed a cross-validation and estimated *M*_*e*_ to range 4000–6100 in Holstein, 2400 in Jersey, and 1800 in Australian Red cattle. The *M*_*e*_ estimates in our study are within these estimates. These discrepancies can be due to the difference in the population because the value of *M*_*e*_ is breed-specific. However, we demonstrated that estimating *M*_*e*_ was independent from heritability. The reliable *M*_*e*_ could not be estimated under the trait with low heritability and polygenic effects. In the condition, it may not be possible to estimate each effect of chromosome segment accurately, and thus inaccurate number of chromosome segment might be estimated under the trait with lower heritability in our study.

In addition to using the results of the empirical accuracies from cross-validation, other methods have been suggested. From the results of the extent of LD in the present population, we estimated an *N*_*e*_ of 101, according to the method of Wientjes et al. [40]. Briefly, *N*_*e*_ *t* generations ago (*N*_*t*_) were obtained using the formula ${N}_t=\left(\frac{1}{r^2}-1\right)/4c$ [41], where *c* = 1/2*t* is the length of the chromosome segment in morgans [42], *r*^*2*^ is the measure of LD over a chromosome segment with length c. Each *N*_*t*_ for *t* values 1–5 was estimated and the mean *N*_*t*_ was defined as *N*_*e*_ in the present population. Applying this *N*_*e*_ value to the equation of Goddard [38], the *M*_*e*_ of 676 was estimated using the equation *M*_*e*_ = 2*N*_*e*_*L*/ *ln* (4*N*_*e*_*L*), where *L* was an assumed genome size of 31.6 M [43]. Wientjes et al. [40] estimated *N*_*e*_ of 123 and *M*_*e*_ of 805 using a Holstein-Friesian cattle population, with which our estimates were comparable. On the other hand, our estimates of *M*_*e*_ using *N*_*e*_ were ^1^/_6_ to ^1^/_3_ of those estimated using the cross-validation results. The *M*_*e*_ value can be either underestimated or overestimated depending on the formula with *N*_*e*_ according to a meta-analysis by Brard & Ricard [44]. Thus, our estimates of *M*_*e*_ derived from *N*_*e*_ might have been underestimated, which in turn, would lead to overestimated accuracy of genomic prediction. To confirm this, we calculated the accuracy of GEBV using Eq. (2) based on the estimated *M*_*e*_ (Fig. S3). Fig. S3 shows that accuracy determined based on *N*_*e*_ seemed overestimated and unrealistic.

A method for estimating *M*_*e*_ using a pedigree relationship matrix (**A**) and a genomic relationship matrix (**G**) between individuals has been proposed [40, 45]. Wientjes et al. [40] estimated a *M*_*e*_ of 837 using **A** and **G** from their study population and it was similar to the *M*_*e*_ of 805 estimated based on the equation of Goddard [38], who used *N*_*e*_. The study by van den Berg et al. [39] found that using both **A** and **G** led to an overestimation of *M*_*e*_, due to the population containing genetically close individuals. However, such overestimation was unlikely to occur in our population because we excluded genetically close individuals from the population.

### Comparison between expected and actual accuracy

We found that the prediction accuracy of the GEBVs for the simulated trait was lower than that for the carcass trait in terms of heritability. This trend became more significant as the size of the reference population increased. Two reasons might account for this finding. One is the definition of accuracy. The accuracy of GEBV is generally a correlation between GEBV and TBV, which is equal to the correlation between GEBV and EBV divided by the correlation between EBV and TBV [7]. Here, the correlation between EBV and TBV was equal to the square root of heritability. However, we used the adjusted phenotype (sum of EBV and residual effect) instead of EBV, because pedigree information was not available. Thus, we defined accuracy of genomic prediction as a correlation between GEBVs and adjusted phenotypes divided by the square root of heritability for carcass traits. Accordingly, for carcass traits with unknown TBVs, accuracy of genomic prediction might be biased using the adjusted phenotypes.

The other is the difference in the QTL distribution between the simulated and carcass traits. Especially for CW, the actual accuracy exceeded the expected accuracy for heritability of 0.5, when the reference population comprised > 5000 animals. Whereas we derived simulated traits from the QTLs following a gamma distribution, a few QTLs with large effects for CW, which accounted for one-third of the total genetic variance, were distributed in specific regions [46]. Moreover, the effects of each QTL were independent in the simulation of phenotypes, and interactions between markers (epistasis effects) were ignored. These considerations might apply not only to CW where QTL positions with large effects are known, but also for REA and BMS, the accuracy of which exceeded that for simulated traits. Although genomic evaluations have not been implemented in Japan for traits such as reproductive performance [47, 48] and feed efficiency [17, 49], we expect that the accuracy of GEBV for such traits would be similar to our simulated traits.

## Conclusion

We conducted a genomic evaluation for simulated traits and carcass traits on a much larger scale in Japanese Black cattle than previous studies. The simulation analysis based on a cross-validation design using real genotypes to account for the extent of LD in this breed revealed that higher heritability and a larger reference population led to improved prediction accuracy of GEBVs, whereas the number of QTLs did not affect accuracy. We developed a deterministic formula based on *M*_*e*_ derived from empirical observations to obtain expected accuracy of GEBV, although estimates of *M*_*e*_ differed by heritability. We found that the expected accuracy of GEBV for a polygenic trait with heritability of 0.1–0.5 could be practical when the reference population comprised > 5000 animals. For carcass traits, we demonstrated that a total of 7000–11,000 animals can be a sufficient size of reference population for genomic prediction.

## Supplementary Information

**Additional file 1: Fig. S1.** Average linkage disequilibrium (*r*^*2*^) values plotted against intermarker distance for all chromosomes. X axis, distance between single nucleotide polymorphisms (SNPs); Y axis, *r*^*2*^ values between SNPs. **Fig. S2.** Accuracy of estimated breeding values (EBVs) for carcass traits with heritability estimates. We calculated EBVs according to Mrode (2005). X axis, number of progenies per candidate bull. Y axis, accuracy of EBV calculated from numbers of progenies and heritability. **Fig. S3.** Expected accuracy of genomic estimated breeding values (GEBVs) for simulated traits based on numbers of independent chromosome segments (*M*_*e*_) estimated from cross-validation findings vs. those from effective population size. X axis, number of animals per reference population. Y axis, expected accuracy of GEBVs for simulated traits with different values of *M*_*e*_ per number of QTLs (nQTL) determined using formula developed herein (black, red, and blue curves) and from effective population size (green curve). Heritability: (a), 0.1; (b), 0.3; (c), 0.5. **Table S1.** The numbers of chromosome segments (*M*_*e*_) estimated by cross-validation from previous studies.

## Data Availability

The datasets analyzed during the present study are not available because it is property of the institutions of the prefectures involved in the present study. A request to the data from this study may be sent to the corresponding author, Masayuki Takeda (m0takeda@nlbc.go.jp).
