# Supplementary material for: Exploring the size of reference population for expected accuracy of genomic prediction using simulated and real data in Japanese Black cattle
Source: BMC Genomics. 2021 Nov 6;22:799. doi: 10.1186/s12864-021-08121-z (PMC8572443; doi:10.1186/s12864-021-08121-z)
Supplement: Supplementary file 1 — Additional file 1: Fig. S1. Average linkage disequilibrium (r2) values plotted against intermarker distance for all chromosomes. X axis, distance between single nucleotide polymorphisms (SNPs); Y axis, r2 values between SNPs. Fig. S2. Accuracy of estimated breeding values (EBVs) for carcass traits with heritability estimates. We calculated EBVs according to Mrode (2005). X axis, number of progenies per candidate bull. Y axis, accuracy of EBV calculated from numbers of progenies and heritability. Fig. S3. Expected accuracy of genomic estimated breeding values (GEBVs) for simulated traits based on numbers of independent chromosome segments (Me) estimated from cross-validation findings vs. those from effective population size. X axis, number of animals per reference population. Y axis, expected accuracy of GEBVs for simulated traits with different values of Me per number of QTLs (nQTL) determined using formula developed herein (black, red, and blue curves) and from effective population size (green curve). Heritability: (a), 0.1; (b), 0.3; (c), 0.5. Table S1. The numbers of chromosome segments (Me) estimated by cross-validation from previous studies. [file 12864_2021_8121_MOESM1_ESM.pptx]

## Slide 1
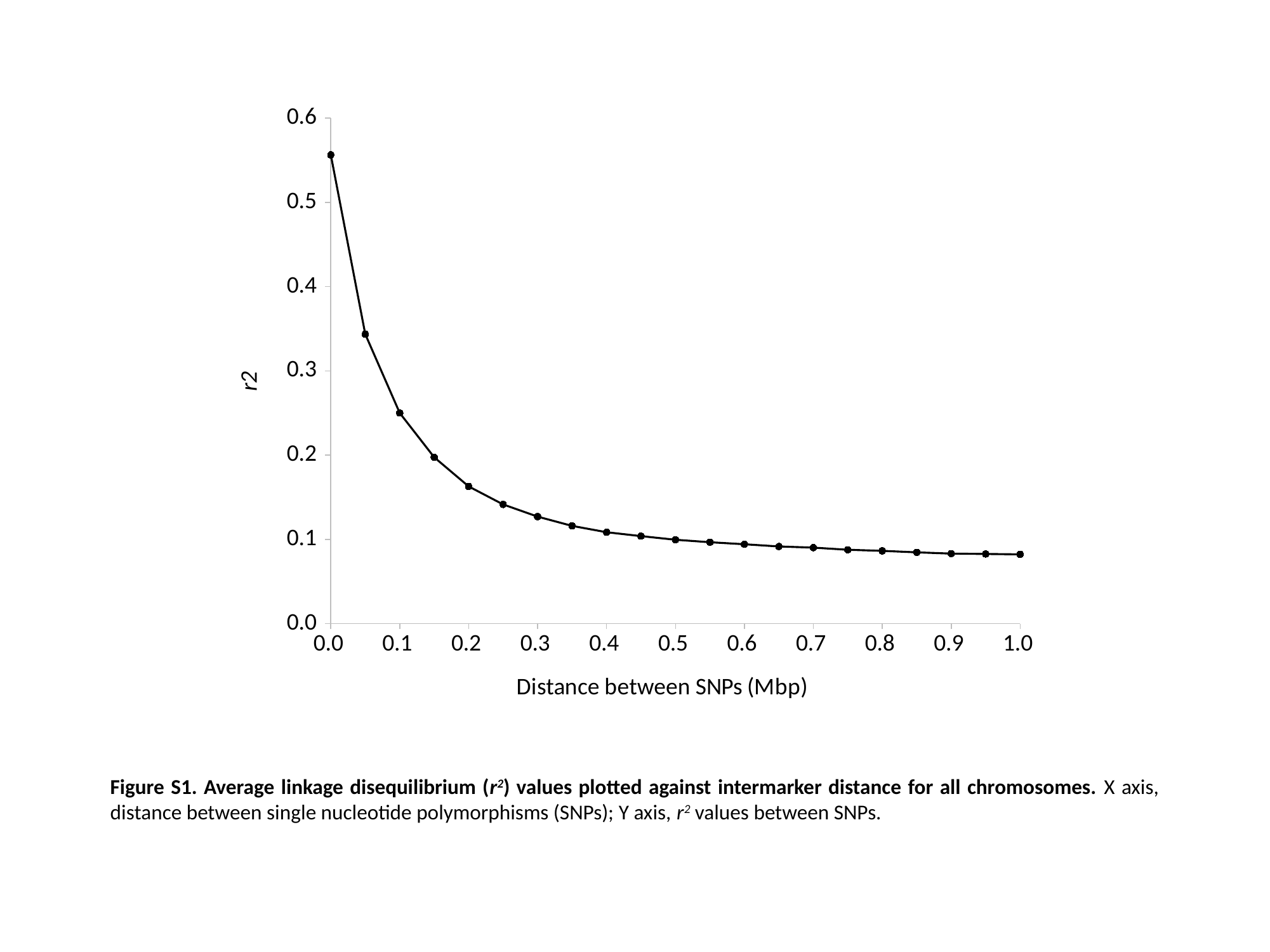

### Chart
| Category | HD |
|---|---|Figure S1. Average linkage disequilibrium (r2) values plotted against intermarker distance for all chromosomes. X axis, distance between single nucleotide polymorphisms (SNPs); Y axis, r2 values between SNPs.

## Slide 2
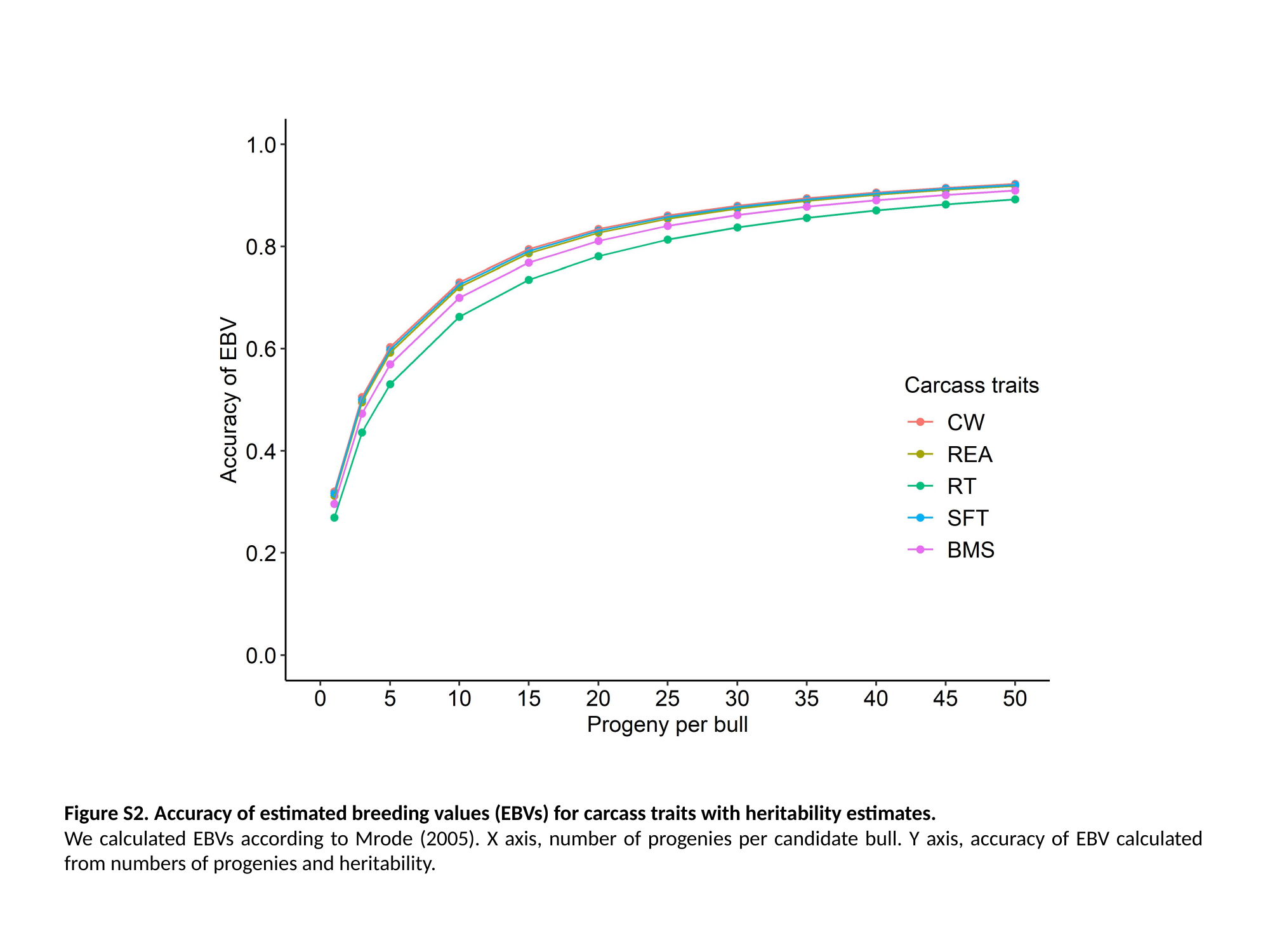

Figure S2. Accuracy of estimated breeding values (EBVs) for carcass traits with heritability estimates.
We calculated EBVs according to Mrode (2005). X axis, number of progenies per candidate bull. Y axis, accuracy of EBV calculated from numbers of progenies and heritability.

## Slide 3
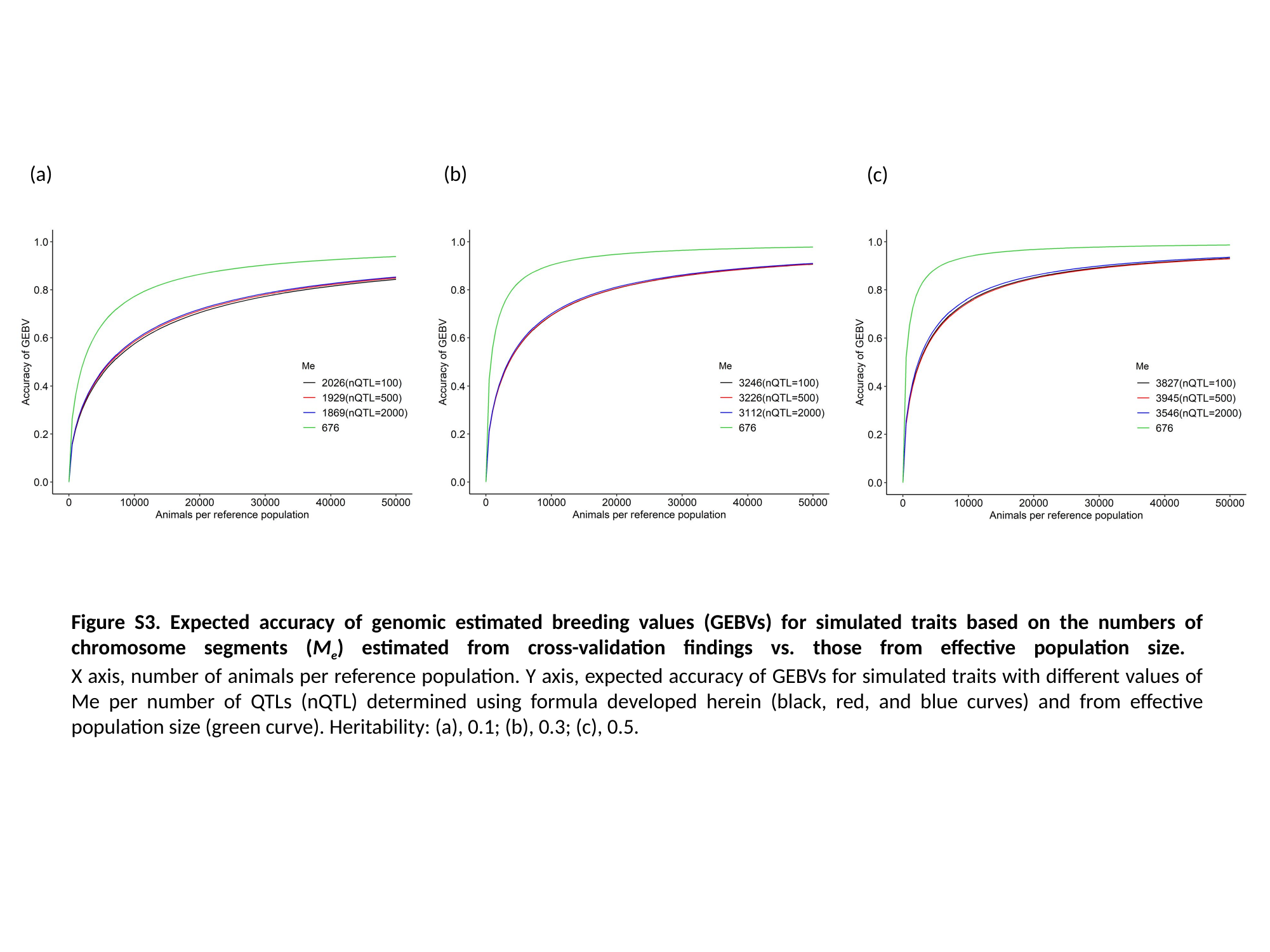

(a)
(b)
(c)
Figure S3. Expected accuracy of genomic estimated breeding values (GEBVs) for simulated traits based on the numbers of chromosome segments (Me) estimated from cross-validation findings vs. those from effective population size.
X axis, number of animals per reference population. Y axis, expected accuracy of GEBVs for simulated traits with different values of Me per number of QTLs (nQTL) determined using formula developed herein (black, red, and blue curves) and from effective population size (green curve). Heritability: (a), 0.1; (b), 0.3; (c), 0.5.

## Slide 4
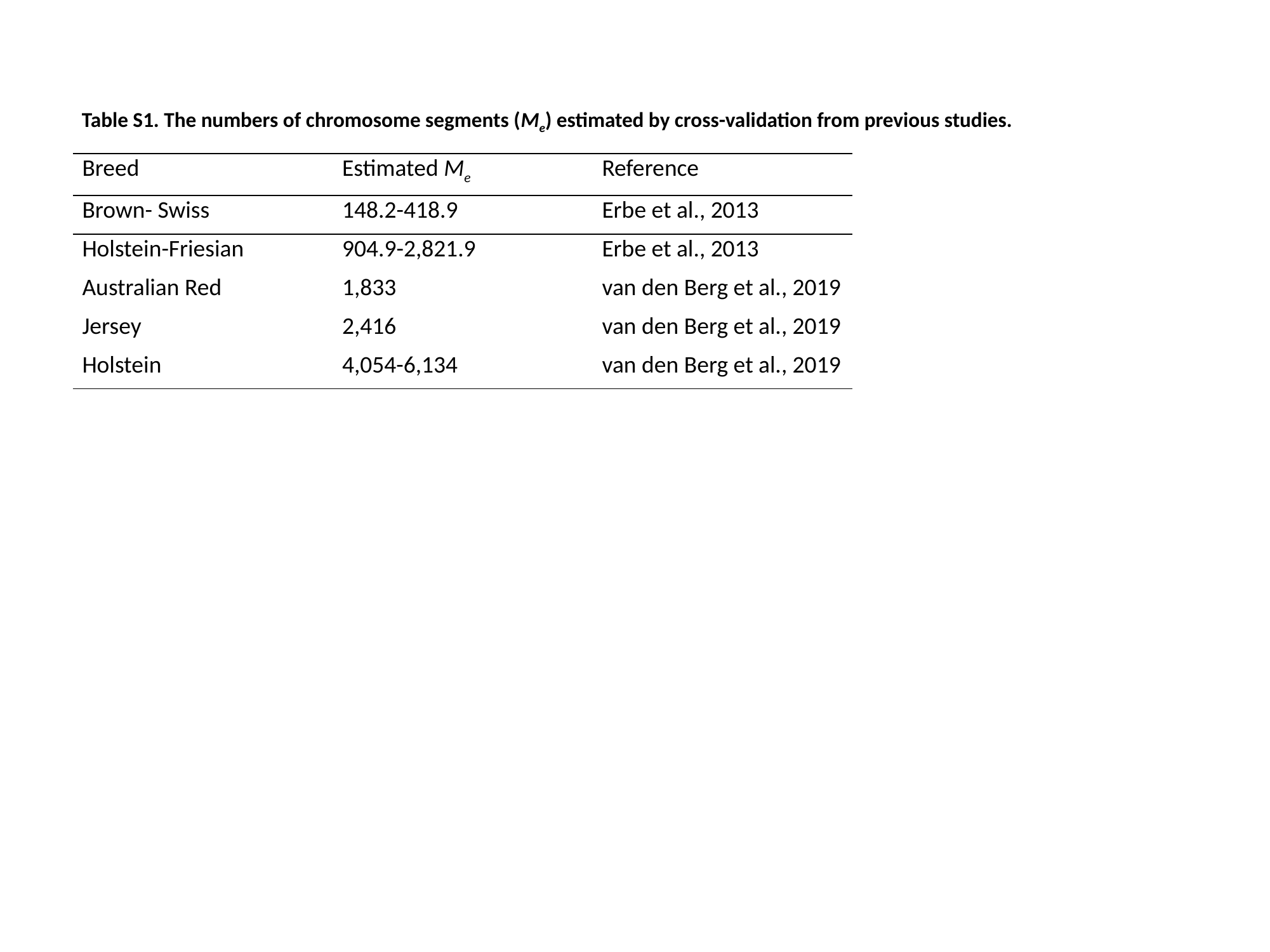

Table S1. The numbers of chromosome segments (Me) estimated by cross-validation from previous studies.
| Breed | Estimated Me | Reference |
| --- | --- | --- |
| Brown- Swiss | 148.2-418.9 | Erbe et al., 2013 |
| Holstein-Friesian | 904.9-2,821.9 | Erbe et al., 2013 |
| Australian Red | 1,833 | van den Berg et al., 2019 |
| Jersey | 2,416 | van den Berg et al., 2019 |
| Holstein | 4,054-6,134 | van den Berg et al., 2019 |
